# Effect of Milling Speed on the Properties of Zirconia Restorations

**DOI:** 10.4317/jced.61231

**Published:** 2024-01-01

**Authors:** Adam J. Wallum, Christopher Raimondi, Wen Lien, Wyeth L. Hoopes, Kraig S. Vandewalle

**Affiliations:** 1 DMD, MS. Maj, USAF, DC. Director, Restorative Dentistry Advanced Education in General Dentistry Residency. AF Postgraduate Dental School. 1615 Truemper St Joint Base San Antonio - Lackland, TX, USA 78236. Uniformed Services University of the Health Sciences, Bethesda, MD, USA; 2 DDS, MS, MS. Lt Col, USAF, DC. Co-Director of Dental Materials Research. USAF Dental Research and Consultation Service. 3650 Chambers Pass, Bldg 3610. Joint Base San Antonio - Fort Sam Houston, TX, USA 78234. Uniformed Services University of the Health Sciences, Bethesda, MD, USA; 3 DMD, MS, MS. Col (ret), USAF, DC. Former Director of Dental Materials Research. USAF Dental Research and Consultation Service. 3650 Chambers Pass, Bldg 3610. Joint Base San Antonio - Fort Sam Houston, TX, USA, 78234. Uniformed Services University of the Health Sciences, Bethesda, MD, 20814, USA; 4 DDS, MS. Lt Col, USAF, DC. Program Director. Advanced Education in General Dentistry Residency. 4102 Pinion Drive. US Air Force Academy, CO, USA 80840. Uniformed Services University of the Health Sciences, Bethesda, MD, USA; 5 DDS, MS. Col (ret), USAF, DC. Air Force Consultant in Dental Research. Advanced Education in General Dentistry Residency. AF Postgraduate Dental School. 1615 Truemper St. Joint Base San Antonio - Lackland, TX, USA 78236. Uniformed Services University of the Health Sciences, Bethesda, MD, USA

## Abstract

**Background:**

The purpose of this study was to evaluate the effect of milling speed on the surface roughness, marginal gap, marginal gap volume, marginal offset, and fracture load of zirconia restorations.

**Material and Methods:**

A mandibular molar #30 typodont tooth was digitally scanned and an ideal crown preparation for a zirconia restoration was digitally created. A single master model die of the crown preparation was milled out of a resin material. The master die was scanned, and a final restoration was designed using the bio-copy feature of the typodont tooth. Ten zirconia restorations were milled (CEREC Primemill, Dentsply Sirona) per each of three milling speeds (super-fast, fine, and extra-fine), sintered, and seated on the master die. Surface roughness, marginal gap, marginal gap volume, and marginal offset were measured using a non-contact profilometer. Then, all restorations were cemented to the dies and loaded to failure in a material testing device. Data were analyzed with one-way ANOVA and Tukey’s post hoc tests per property (alpha=0.05).

**Results:**

Super-fast milling speed resulted in restorations with statistically significant greater surface roughness and marginal gap volume compared to fine and extra-fine milling speeds. No significant difference in marginal gap, marginal offset, and fracture load were found based on milling speed.

**Conclusions:**

Zirconia restorations milled at slower speeds may result in similar or slightly better properties compared to super-fast speed.

** Key words:**Milling speed, properties, zirconia restorations.

## Introduction

New ceramic materials provide an excellent balance of strength and esthetics that have driven a steep rise in popularity among dental providers. Zirconia restorations may be produced using computer-aided design and computer-aided manufacturing (CAD/CAM) technologies in either a laboratory or chairside clinical setting. Compared to laboratory procedures, a chairside workflow allows the clinician to design and manufacture the restoration utilizing same-day dentistry. Historically, milled zirconia restorations could not practically be considered a single appointment restoration because of the long sintering procedures. The advent of high-speed sintering allowed the fabrication of zirconia restorations in as little as 15 minutes compared to conventional sintering which could take as long as 6-8 hours (1-4). A systematic reviews of laboratory studies determined that mechanical and precision results were similar or better when high-speed methods were used for millable zirconia materials (4). More recently, the possibility of super-fast milling has been introduced to the market to further expedite the chairside process. Dentsply Sirona (Charlotte, NC, USA) recently released a milling unit (CEREC Primemill) that reportedly has the fastest milling time available on the market and can produce zirconia restorations in as little as five minutes in super-fast mode. The milling unit has 3 options - “super-fast”, “fine”, or “extra-fine” - for milling of the zirconia material used in this study (5).

The machining process inherently produces cracks, chipping, subsurface damage, and residual stresses in ceramic materials (6,7). No research has been published evaluating the effect of super-fast milling speeds on the properties of ceramic restorations. The purpose of this study was to evaluate the effect of different milling speeds (super-fast, fine, or extra-fine) on the surface roughness, marginal gap, marginal gap volume, marginal offset, or fracture load of zirconia restorations. The null hypothesis was that there would be no difference in the properties of a zirconia crown restoration based on milling speed.

## Material and Methods

An intact typodont tooth (#30) (Kilgore 200, Kilgore International, Coldwater, MI, USA) was scanned using a chairside acquisition unit (CEREC Primescan, Dentsply Sirona). An ideal crown preparation for a zirconia restoration was designed in exocad (exocad GmbH, Darmstadt Germany) following the manufacturer’s recommendation for a 4 mol% yttria partially stabilized zirconia (4Y-PSZ) material (Katana STML, Kuraray Noritake, Tokyo, Japan) for a posterior crown restoration: 1mm occlusal reduction, at least 4mm preparation height with axial convergence of 10%, 1 mm uniform axial reduction and 1mm wide circumferential shoulder finish line with rounded internal angles. The design was exported and saved in standard tessellation language (STL) format as the “master” file for milling duplicate tooth preparation dies. The STL file was imported into milling software (iCAM V5, I-Mes, iCore, Eiterfeld, Germany). Thirty model specimens of the preparation were milled in a five-axis milling unit (I-Mes, iCore) using a fiberglass and resin material (Trinia, Bicon, Boston, MA, USA) with an elastic modulus similar to dentin (8).

One single master die of a model specimen was scanned with the CEREC Primescan acquisition unit. A virtual restoration was designed following the contour of an unprepared typodont tooth (#30) using the Biogeneric Copy feature in the CEREC Primescan software (version 5.2, Dentsply Sirona). The restoration spacer was standardized at 100um. Thirty identical crowns (n=10 per group) were milled from the zirconia blocks (size 12Z) utilizing new milling burs per group on a calibrated CEREC Primemill unit. Three groups (n=10) based on milling speeds of super-fast (SF), fine (F), and extra-fine (EF) were utilized in this study. The milling time for the crown in each group was recorded. All crowns were sintered dry in a zirconia furnace (Programat S1 1600, Ivoclar Vivadent, Schaan, Liechtenstein) and steam cleaned (7000CJ, Reliable, North York, ON, Canada) following the manufacturer’s instructions.

**Figure 1:**
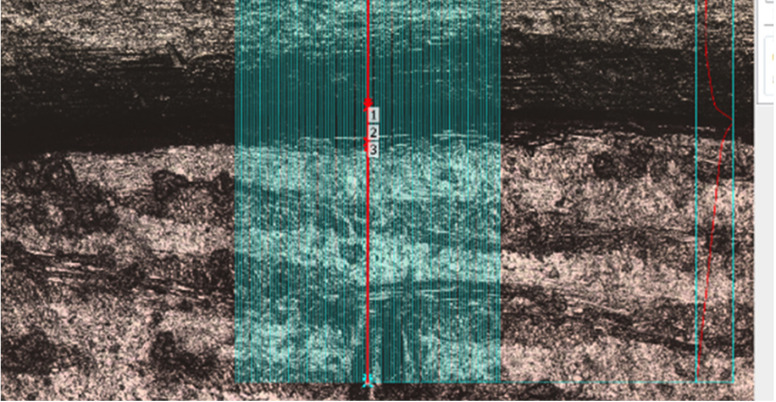
Magnified view depicting location of margin measurements between crown and die. Fifty measurements were completed in 1µm increments utilizing the software. The measurements were averaged by the software.

A custom mounting jig was fabricated to orient and seat each restoration accurately and consistently on the single master die. Surface roughness, marginal gap, marginal gap volume per 500-micron length, and marginal offset were measured using a non-contact profilometer (3D Laser-Scanning Confocal Profilometer, Keyence, Itasca, IL, USA) and then analyzed using its proprietary software. All parameters were measured once on the buccal, lingual, mesial, and distal surfaces for each crown (n=40). A small notch was created on the master die away from the margin on all four surfaces to standardize the measurement location. A magnified view depicting location of margin measurements between crown and die is shown in Figure 1. Surface roughness (Sa) was measured 2mm coronal to the margin.

The specimens were polished using polishing wheels (Dialite ZR, Brasseler, Savannah, GA, USA). The polishing was done by one operator with standardized polishing wheels to the same level of polish assessed visually. New polishers were used for each group. The thirty individual crowns were then cemented to the thirty preparation model specimens using a self-adhesive resin cement (Panavia SA, Kuraray) following the manufacturer’s instructions. The crowns were cemented with finger pressure by one operator. An initial 2-second polymerization per surface was completed using an LED light-curing unit (Valo Grand, Ultradent Products, South Jordan, UT, USA). The excess cement was removed from the margin and then further polymerized with the light-curing unit for 20 seconds from the occlusal, facial, and lingual surfaces of each crown. Next, the specimens were individually mounted with denture acrylic (Vitacrylic, Fricke Dental, Streamwood, IL, USA) inside a polyvinyl chloride (PVC) pipe using a custom mounting jig to ensure standardized orientation. Then the specimens were incubated for 24 hours in a lab oven (Model 20, GC Lab, Quincy Lab, Chicago, IL, USA) in distilled water at 37 degrees C.

The specimens were then placed into a vise fixture on a universal testing machine (Model #5943, Instron, Norwood, MA, USA) so that the specimen’s occlusal surface was at a 90-degree angle from the testing fixture. A stainless-steel rod, six millimeters in diameter was used to load the central fossa. Specimens were loaded at a rate of 1.0 mm per minute until failure was reached. The fracture load was recorded in Newtons. A mean and standard deviation for all properties were determined for all three groups. Data were initially analyzed with a Shapiro Wilk test for normality and Levene’s test for homogeneity and subsequently one-way ANOVA and Tukey’s post hoc tests to determine any differences in surface roughness, marginal gap, marginal gap volume per 500-micron length, marginal offset, and fracture load of the zirconia crowns based on milling speed (alpha=0.05). Failure mode was determined by visual examination to determine if the failure was Type 1 - fracture with greater than 50% of the crown remaining; Type 2 - fracture with 50% or less of the crown remaining; Type 3 - fracture and loss of the entire crown; Type 4 - fracture of the crown and die material; Type 5 - severe crown and die material fracture (catastrophic failure).

## Results

The milling times recorded were 5 minutes 31 seconds for super-fast, 17 minutes 59 seconds for fine, and 19 minutes 33 seconds for extra-fine settings. The data were found to be normally distributed and homogeneous in variance (*p*>0.05). The values of each tested property based on milling speed are shown in Table 1. Super-fast milling speed resulted in the greatest surface roughness (3.8 ± 0.7 µm) and it was significantly rougher than fine (2.8 ± 0.4 µm, *p*<0.001) or extra-fine (2.9 ± 0.3 µm, *p*<0.001) milling speeds. The greatest marginal gap was found with super-fast milling speed (88.3 ± 30.3 µm), but it was not significantly different from fine (74.6 ± 22.0 µm, *p*=0.088) or extra-fine (74.8 ± 32.6 µm, *p*=0.093) milling speeds. Super-fast milling speed produced significantly greater marginal gap volume per 500 µm length (0.46 ± 0.23 nL) than fine (0.31 ± 0.22 nL, *p*=0.004) or extra-fine (0.32 ± 0.19 nL, *p*=0.006) milling speeds. The greatest marginal gap offset was found with super-fast milling speed (65.2 ± 43.4 µm), but it was not significantly greater than fine (48.1 ± 27.2 µm, *p*=0.067) or extra-fine (48.5 ± 28.7 µm, *p*=0.076) milling speeds. The greatest fracture strength was found with fine milling speed (5713.8 ± 527.8 N), but it was not significantly different from super-fast (5292.8 ± 472.8 N, *p*=0.127) or extra-fine (5329.5 ± 387.4 N, *p*=0.175) milling speeds. There were no significant differences (*p*>0.175) between fine and extra-fine milling speeds for any of the tested properties. The mode of fracture was primarily Type 2 (fracture with 50% or less of the crown remaining) for all three groups. The super-fast specimens had 90% Type 2 failures with 10% Type 3 (fracture and loss of the entire crown). The fine specimens had 80% Type 2 failures with 20% Type 3. The extra-fine specimens had 100% Type 2 failures.

**Table 1:**
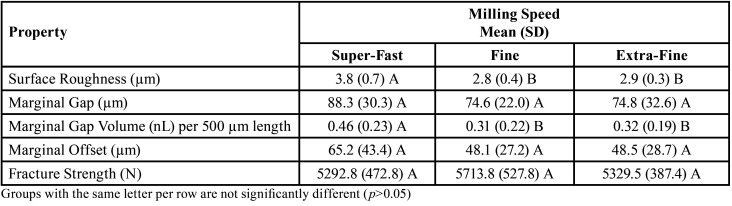
Values of each property tested based on milling speed.

## Discussion

The demand for same day dentistry has pushed technology to be more convenient and rapid. Software advances have improved the speed of designing restorations. The speed-sintering process for zirconia restorations has allowed zirconia to become an option for same-day dentistry without a significant sacrifice in physical properties (1-4). Super-fast milling is the most recent innovation. The zirconia crown restorations in this study were milled in approximately 5.5 minutes in super-fast mode versus 18 minutes for fine and 19.5 minutes for extra-fine settings, resulting in 69.4% and 71.8% reduction in milling time, respectively. Actual milling times will vary depending on the size and type of restoration. As determined in this study, differences were found in some properties based on milling speed; therefore, the null hypothesis was partially rejected. Statistically significant differences were found for surface roughness and marginal gap volume between super-fast milling and fine/extra-fine milling. No significant differences were found between the groups for marginal gap, marginal offset, and fracture load.

**Figure 2:**
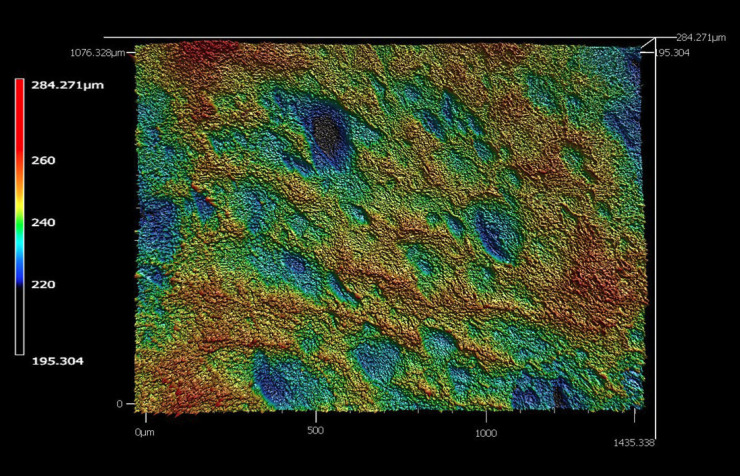
Representative topographical surface roughness images for super-fast (2), fine (3), and extra-fine (4) milling speeds.

**Figure 3:**
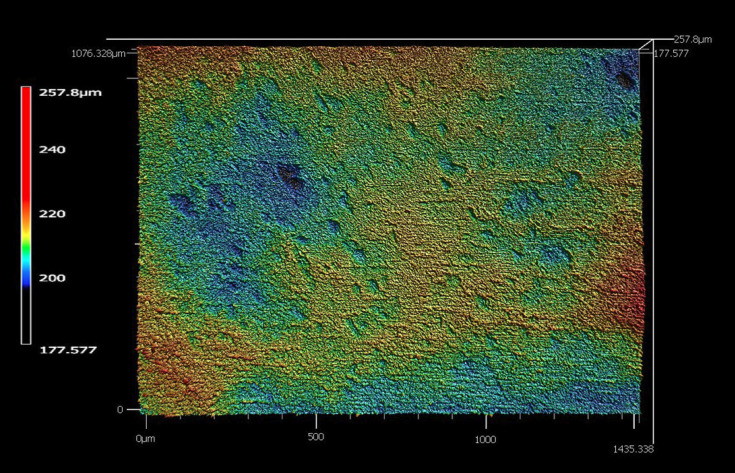
Representative topographical surface roughness images for super-fast (2), fine (3), and extra-fine (4) milling speeds.

**Figure 4:**
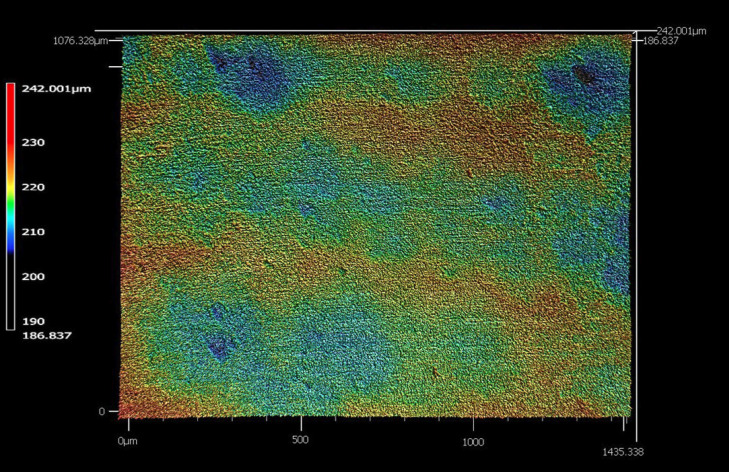
Representative topographical surface roughness images for super-fast (2), fine (3), and extra-fine (4) milling speeds.

Topographical images produced by the confocal profilometer software revealed stark differences in surface roughness between super-fast, fine, and extra fine samples (Figs. 2-4). The super-fast specimens had a distinct grooved pattern that appeared to be created by the exclusive use of the 2.5 ZrO2 CS bur on the external surface. Surface roughness (Sa) was utilized for this study and is defined as a parameter that measures the finely spaced micro-irregularities and topographical textures such as roughness, waviness, and form on a material’s surface (9). The cameo surface roughness of a restoration may have a direct impact on the development of biofilm. Biofilm can be directly responsible for the development of secondary caries and periodontal disease (10-13). Surface roughness can be reduced by finishing and polishing. A rougher surface will require more time chairside to polish the restoration before insertion. Polishing can be completed in either the pre- or post-sintered state for zirconia restorations.

Although there was a trend for super-fast milling to result in means for all three marginal interface measurements that were greater than that of fine and extra-fine milling, only the marginal gap volume was significantly greater for super-fast milling. No definitive value for a clinically acceptable margin gap has been defined, but a range of 39 µm to 150 µm has been stated in previous literature (7). The marginal gap for all three groups was within the accepted range, therefore, the differences in the marginal interface values between the three milling speeds may not be clinically significant.

The intaglio surfaces of ceramic restorations have been shown to concentrate tensile forces that can initiate fracture in the defects on this surface (6). In addition, the cervical restoration margin demonstrates importance in fracture initiation (14). A direct correlation has also been established between surface roughness or surface damage and a reduction in flexural load (15). However, super-fast milling did not reduce the fracture load of the cemented crown restorations evaluated in this study. The fracture loads reported in this study were greater than the estimated maximum clinical chewing force of 965 N (16). The restorations were so strong, that when they failed, the zirconia ceramic shattered, leaving less than 50% of the remaining crown intact.

The three separate milling speeds of the CEREC Primemill utilize different tools during milling. Variations in the tools allow for faster or more detailed milling. As part of the software program, tool integrity was monitored during milling of the specimens. Super-fast milling used tools at a rate 2.85 and 2.44 times faster than fine and extra-fine milling, respectively. The greater tool rate for super-fast milling may add a significantly higher cost to the milling procedure compared to slower milling speeds.

Limitations to this study include the use of only one zirconia material, crown type, and milling device. Additionally, the crowns were cemented to model specimens made of a fiberglass and resin material and not to natural teeth. Future studies should examine the clinical differences between the use of super-fast versus fine and extra fine milling speeds.

## Conclusions

Super-fast milling speed resulted in restorations with significantly greater surface roughness and marginal gap volume compared to fine and extra-fine milling speeds. No significant difference in marginal gap, marginal offset, or fracture load were found based on milling speed. Zirconia restorations milled at slower speeds may result in similar or slightly better properties compared to super-fast speed.
